# Chagas Cardiomyopathy: Usefulness of EKG and Echocardiogram in a Non-Endemic Country

**DOI:** 10.1371/journal.pone.0157597

**Published:** 2016-06-16

**Authors:** Adrián Sánchez-Montalvá, Fernando Salvador, José Rodríguez-Palomares, Elena Sulleiro, Augusto Sao-Avilés, Sílvia Roure, Lluís Valerio, Arturo Evangelista, Israel Molina

**Affiliations:** 1 Tropical Medicine Unit, Infectious Disease Department. PROSICS (International Health Program of the Catalan Health Institute), Hospital Universitario Vall d’Hebron, Universitat Autònoma de Barcelona, Barcelona, Spain; 2 Cardiology Department, Cardiac Imaging Department, Hospital Universitario Vall d’Hebron, Universitat Autònoma de Barcelona, Barcelona, Spain; 3 Microbiology Department, Hospital Universitario Vall d’Hebron, Universitat Autònoma de Barcelona, Barcelona, Spain; 4 North Metropolitan International Health Unit, PROSICS (International Health Program of the Catalan Health Institute), Universitat Autònoma de Barcelona, Barcelona, Spain; Hospital Universitario LA FE, SPAIN

## Abstract

**Background:**

Chagas disease (CD) is a major cause of cardiomyopathy in Latin America, and migration movements have now spread the disease worldwide. However, data regarding Chagas cardiomyopathy (CC) and the usefulness of echocardiography in non endemic countries are still scarce.

**Methods and results:**

We selected 485 patients in the chronic phase of CD from two Spanish settings. Data from physical examination, electrocardiogram (EKG), x-ray, and two dimensional transthoracic echocardiogram were recorded. *Trypanosoma cruzi* DNA was assessed by PCR in peripheral blood. Patients were stratified according to the Kuschnir classification and a combination of echocardiogram and electrocardiogram findings. Patients mainly came from Bolivia (459; 94.6%). One hundred and forty three patients (31.5%) had at least one electrocardiogram abnormality. Twenty seven patients (5.3%) had an abnormal echocardiography. Patients with abnormal echocardiography were older (47 (IQR 38–57) years vs 41 (IQR 38–57) years); p = 0.019) and there was a greater proportion of males (66.7% vs 29.7%); p<0.001). Among echocardiographic variables, diastolic dysfunction was associated with poor cardiac status. In the multivariate analysis, abnormal EKG and gender were associated with abnormal echocardiography. Echocardiography may be spared for males under 30 and females under 45 years old with normal EKG as the likelihood of having an abnormal echocardiography is minimal. Association between *T*. *cruzi* DNA in the peripheral blood and cardiac involvement was not observed.

**Conclusion:**

CC rates in the studied population are low. Age and sex are important determinants for the development of CC, and with the EKG should guide echocardiogram performance.

## Introduction

Chagas disease (CD) is an infection caused by the haemoflagellate protozoan *Trypanosoma cruzi*. It is a major health issue in Latin America, where the disease is endemic. It affects 8 million people worldwide and 25 million remain at risk.[1] CD causes 10000 casualties every year. The global costs are estimated at 7.19 billion dollars per year, mainly due to lost productivity from cardiovascular complications.[2] CD is considered a neglected disease, as poverty is a significant determinant in contracting the disease and it has received little attention from research. Lately, migratory flows have spread the disease worldwide, the majority of cases outside the endemic countries being found in The United States of America and Spain. It is estimated that approximately 43000 migrants infected with *T*. *cruzi* are living in Spain.[3]

After an acute phase characterized by mild symptoms, *T*. *cruzi* infected patients enter a chronic phase that usually lasts decades. According to clinical symptoms, physical signs and additional diagnostic tests, the patients are classified in the indeterminate, cardiac or digestive form. In the indeterminate form, patients are asymptomatic, have a normal electrocardiogram (EKG) and no evidence of cardiomegaly. Cardiac involvement is variable among regions; however, 40–60% of the patients will develop cardiac complications.[4,5] Most patients are diagnosed during the chronic phase. Although high parasitemia exists during the acute phase of the disease, severe myocardial involvement is rare, but fatal if present. In the chronic phase parasitemia is present in approximately 60% of the patients.[6] However, whether *T*. *cruzi* polymerase chain reaction (PCR) is associated with progression to cardiac involvement is still under discussion.[7,8]

Death in CD patients occurs mainly because of electrical disturbances or cardiac failure related to dilated cardiomyopathy.[9] Once cardiac damage is presented, the mortality rate for the next 7.9 years is 30.7%,[10] outranging the mortality of other causes of heart failure.[11]

Several criteria exist to define Chagas cardiomyopathy (CC). Classical criteria were based on EKG abnormalities, thorax x-ray and symptoms.[12] EKG readings and symptoms are not always easy to interpret and can lead to the misclassification of patients. To avoid mistakes in EKG, reading protocols advising how to read EKG in CC have been performed;[13] however, difficulties may persist. New criteria also include echocardiographic parameters to define CC.[14] Patients classified as having CC have a different mortality risk depending on which parameter they have altered.[10]

The objectives of our study are: a) to describe the EKG and echocardiography abnormalities in CC in a non-endemic area, b) to compare different CC classification methods, c) to assess the usefulness of age, gender and EKG to predict echocardiography abnormalities and d) to assess the association between PCR positivity and CC.

## Methods

### Study population

Consecutive CD patients attending the Tropical Medicine Unit of the Vall d’Hebron University Hospital (Barcelona) between November 2007 and January 2014 and the North Metropolitan International Health Unit (Santa Coloma) between November 2009 and January 2014 were assessed with a full medical history and demographic characteristics, physical examination, 12-lead electrocardiography, chest x-ray and a two-dimensional transthoracic echocardiogram (2D-TTE).

Inclusion criteria included a minimum age of 17 years old, confirmed diagnosis of CD and having a 2D-TTE performed. Patients with diabetes mellitus, hypertension, alcoholism, cardiac ischemic history or other cardiovascular diseases were excluded. Patients previously treated with benznidazole or nifurtimox were also excluded.

Diagnosis of CD was based on two positive serological enzyme-linked immunosorbent assay (ELISA) tests, one with recombinant antigen (Bioelisa Chagas, Biokit, Lliçà d’Amunt, Spain) and the other with crude antigen (Ortho *T*. *cruzi* ELISA, Jonhson & Jonhson, High Wycombe, United Kingdom). If discordant results were obtained, sera was tested by a in-house Western Blot method using a lysate from *T*. *cruzi* epimastigote to confirm the diagnosis.[15] Furthermore, *T*. *cruzi* DNA was assessed in the peripheral blood by qualitative PCR before initiating treatment with benznidazole. PCR assay was performed according to Piron et al[16]. *T*. *cruzi* DNA is not currently used in the diagnosis process of patients with CD, but it is used with research purposes. The *T*. *cruzi* DNA assessment was only performed in the patients attended at the Tropical Medicine Unit of the Vall d’Hebron University Hospital.

### Cardiac assessment

Patients were asked for symptoms of cardiac failure. New York Heart Association (NYHA) staging was recorded in all patients. Cardiomegaly was considered when the cardiac silhouette in the thorax X-ray was bigger than 0.5 of the amplitude of the thorax.

All 12 lead EKG recordings lasted between 10 and 30 seconds and were performed after at least 5 minutes of inactivity. The modified Minnesota code adapted for CD was used to evaluate electrocardiographic abnormalities.[13] EKGs were read by two well-trained physicians; if any disagreement arose, a final decision was reached by consensus between both. EKG abnormalities considered to be attributed to CD were: isolated right bundle branch block, isolated left anterior fascicular block, isolated left posterior fascicular block, left bundle branch block, premature ventricular contraction, Q waves, ST-T changes, first or second degree atrioventricular (AV) block, complete AV block, low QRS voltage, sinus bradycardia <50 beats per minute, atrial fibrillation or flutter, and pacemaker rhythm.

All patients underwent a comprehensive grey-scale second-harmonic 2D-TTE performed by an experienced echocardiographer using high-quality commercially available ultrasound systems (Vivid 7 and Vivid E9 [General Electric Vingmed, Horten, Norway]). Left ventricular, aortic and left atrial dimensions were obtained using the M mode convention. The ejection fraction (EF) was calculated using the biplane Simpson's method, and wall motion was assessed in the parasternal short-axis and the apical two-chamber, four-chamber, and long-axis views. Colour flow mapping and pulsed and continuous wave Doppler recordings were obtained in all subjects. Diastolic function patterns were divided into normal, impaired relaxation (stage I), pseudonormal (stage II) and restrictive pattern (stage III). Normal reference values were considered according to the guidelines of the European Association of Cardiovascular Imaging.[17]

There are many classifications to stratify CC.[12,18,19] In our study patients with CD were classified according to the EKG, NYHA, and thorax x-ray results, as defined by Kuschnir.[12] In this classification abnormalities of the EKG that have been described above or cardiac enlargement by thorax-ray or signs and symptoms of heart failure are considered as definitive CC. Alternatively, patients were also classified in three groups (echostage classification) according to the EKG and echocardiographic findings. Patients in group 1 had normal EKG and normal echocardiography, patients in group 2 had EKG abnormalities and normal echocardiography and patients in group 3 had impaired echocardiography, irrespective of EKG findings. Echocardiography was considered abnormal if EF <50% or left ventricular end diastolic diameter (LVEDD) > 55mm or a segmental contractile impairment was present.

### Statistical analysis

Data were analyzed with IBM^®^ SPSS^®^ Statistics (v.21.0.0.0) software. Median and interquartile range (IQR) were calculated for quantitative variables, while frequencies and percentages were calculated for qualitative variables. Univariate analysis was performed using ANOVA and Kruskall-Wallis for quantitative variables and Chi-square test or Fisher’s test for qualitative variables. Statistically significant variables within the univariate analysis were then analyzed in a multivariate logistic regression test if they were considered clinically relevant. Additionally, patients were divided according to pretreatment *T*. *cruzi* PCR results and association with defined variables was performed. Tests were considered significant when the p-value was < 0.05.

We selected easily obtainable criteria like gender, age and any EKG alteration, and evaluated sensitivity, specificity and positive and negative predictive values as markers of regional contractile impairment, low EF, and high LVEDD. A receiver operating characteristic (ROC) curve was depicted for the age according to gender to obtain the best cutoff point.

### Ethical considerations

The study protocol was approved by the Ethical Review Board of Vall d’Hebron Hospital (Barcelona, Spain) and procedures were carried out in accordance with the ethical standards laid down in the Helsinki Declaration as revised in 2000. Given the retrospective nature of the study and the number of patients involved a waiver was agreed by the Ethical Review Board. All procedures of the study were done as standard clinical practice. The information of the patients was anonymized and de-identified prior to analysis.

## Results

A total of 485 out of 771 patients fulfilled the inclusion criteria. Three hundred and seventy five (77.32%) patients were from the Tropical Medicine Unit of the Vall d’Hebron University Hospital and 110 (22.68%) patients were from the North Metropolitan International Health Unit. Patients chiefly came from Bolivia (459 patients, 93.6%) with 9 (1.9%) from Argentina (other nationalities are shown in Table 1). The median age was 39 (IQR 31–46.5) years old. One hundred and fifty four (31.8%) patients were male. The mean time of residence in the country before diagnosis was 6 (IQR 4–7) years. Other baseline characteristics are shown in Table 1. Most of the patients were classified as Kuschnir stage 0 or I. Only 9 (2.6%) patients had heart failure and were categorised as Kuschnir stage III.

**Table 1:** Baseline characteristics of the study population (n = 485).

Age (years)	39 (31–46.5)
Gender (male)	154 (31.8%)
BMI	26.87 (24.43–30.25)
Years in Spain before diagnostic	6 (4–7)
Nationality	
Bolivia	459 (94.6%)
Argentina	9 (1.9%)
Ecuador	5 (1%)
Paraguay	3 (0.6%)
Honduras	2 (0.4%)
Venezuela	2 (0.4%)
Others	3 (0.6%)
*Trypanosoma cruzi* PCR before treatment (positive)	128 (42.2%)
**Cardiac characteristics**	
Cardiomegaly	16 (4.2%)
Heart failure	9 (1.8%)
NYHA I	4 (0.8%)
NYHA II	4 (0.8%)
NYHA III	1 (0.2%)
NYHA IV	0
Pacemaker carrier	6 (1.2%)
Abnormal EKG	143 (31.5%)
Abnormal echocardiography	27 (5.6%)
Kuschnir classification	
0	233 (67.0%)
I	98 (28.2%)
II	8 (2.3%)
III	9 (2.6%)
Echostage classification	
Normal EKG and normal echocardiography	302 (66.4%)
Abnormal EKG and normal echocardiography	126 (27.7%)
Abnormal echocardiography	27 (5.9%)
Chagas cardiomyopathy by EKG or echocardiography	
Normal EKG and echocardiography	302 (66.4%)
Abnormal EKG or echocardiography	153 (33.6%)

**Note:** categorical data are shown as total number (frequencies) and quantitative data are shown as median (interquartile range). BMI: body mass index; PCR: Polymerase Chain Reaction; EKG: electrocardiogram.

Nearly 32% of the patients had at least one alteration on the EKG related to CC. The most frequent EKG findings were left anterior fascicular block, right bundle branch block, and bradycardia (Table 2). Only one patient presented with a left bundle branch block. There were 6 (1.2%) patients with pacemakers. More than one EKG abnormality was present in 45 (9.9%) patients.

**Table 2:** EKG findings in the study population (n = 453).

Heart rate <50bpm	23 (5.1%)
Atrial fibrillation	3 (0.7%)
Atrial extrasystole	5 (1.1%)
First degree block	16 (3.5%)
QTc impairment	15 (3.3%)
Right bundle branch block	27 (6%)
Anterior hemiblock	80 (17.8%)
Posterior hemiblock	3 (0.7%)
Left bundle branch block	1 (0.2%)
Ventricular extrasystole	2 (0.4%)
Mobitz I	0
Mobitz II	0
Third degree block	1 (0.2%)
Left ventricular hypertrophy	3 (0.7%)
Low voltage	9 (2%)
T-ST alterations	9 (2%)
Q wave	2 (0.4%)

**Note:** categorical data are shown as total number (frequencies). QTc: Corrected QT Interval.

Left ventricular systolic dysfunction was found in 11 (2.3%) patients. Mild or Moderate dysfunction, defined as EF between 30% and 50%, was present in 9 (72.72% of the patients with left ventricular systolic dysfunction) patients, and 2 patients showed EF below 30%. The main parameters of the echocardiographic data are depicted in Table 3.

**Table 3:** Baseline echocardiographic characteristics of the study population n = 485.

Left ventricular end-diastolic diameter >55mm n = 484	16 (3.3%)
Left ventricular end-systolic diameter >40mm n = 468	9 (1.9%)
Septum thickness >10mm n = 481	122 (25.4%)
Posterior wall thickness >10mm n = 481	72 (14.9%)
Left atrium diameter >40mm n = 464	96 (20.7%)
LVEF < 50% n = 484	11 (2.3%)
Segmental contractile impairment n = 485	10 (2.1%)
TAPSE <16mm n = 376	4 (1.1%)
Deceleration time <120ms n = 311	7 (2.3%)
Pulmonary arterial pressure >40mmHg n = 257	3 (1.2%)
Pulmonary acceleration time <100ms n = 356	14 (3.9%)
Diastolic dysfunction n = 476	
Normal diastolic function	373 (78.4%)
Impaired relaxation	97 (20.4%)
Pseudonormal	6 (1.3%)
Reversible restrictive	0
Fixed restrictive	0
Aneurysm n = 485	10 (2.1%)
Global hypocontractibility n = 485	1 (0.2%)
Interventricular septum anomalous movement n = 485	5 (1%)

**Note:** categorical data are shown as total number (frequencies). LVEF: left ventricular ejection fraction; TAPSE: tricuspid annular plane systolic excursion.

Eight patients, six of whom were male, had normal EKG, while the echocardiography was compatible with CC. Two patients had regional contractile impairments, four had enlargement of the left ventricle and two had an EF between 45–50%.

Patients with abnormal echocardiography (define by define by EF <50% LVEDD > 55mm or segmental contractile impairment) were older (47 years (IQR 38–57) vs 41 years (IQR 35–50) p = 0.019) and more likely to be male (66.7% vs 29.7%; p<0.001) than patients with normal echocardiography. Most of these patients (69.2%) had an abnormal EKG compared with 29.2% of the patients with normal echocardiography (p<0.001. Further, patients with more EKG abnormalities presented abnormal echocargraphyin a higher proportion. In the multivariate model (Table 4), maleness and abnormal EKG were strong predictors of abnormal echocardiography.

**Table 4:** EKG abnormalities by abnormal echocardiography (define by EF <50% LVEDD > 55mm or segmental contractile impairment).

	Abnormal echocardiography n = 27	Normal echocardigraphy n = 458	P	Multivariate analysis
Sinus rhythm	21 (84%)	426 (99.5%)	<0.001	
Right bundle branch block*	5 (21.7%)	22 (5.2%)	0.001	
Anterior hemiblock*	7 (30.4%)	73 (17.1%)	0.155	
Posterior hemiblock*	1 (4.3%)	2 (0.5%)	0.146	
Left bundle branch block*	0	1 (0.2%)	0.99	
Ventricular extrasystoles*	1 (4.3%)	1 (0.2%)	0.1	
Third degree block†	1 (4.2%)	0	0.053	
Low Voltage*	2 (8.7%)	7 (1.6%)	0.072	
Pacemaker weaver	4 (14.8%)	2 (0.4%)	<0.001	
QTc impairment*	2 (8.7%)	13 (3.1%)	0.142	
QRS impairment*	5 (21.7%)	25 (5.9%)	0.003	
Prolonged QRS or pacemaker	8 (30.8%)	26 (6.1%)	<0.001	
Bradycardia*	5 (21.7%)	18 (4.2%)	<0.001	
First degree block	2 (8%)	14 (3.3%)	<0.001	
ST-T abnormalities	1 (4.2%)	6 (1.4%)	0.320	
Q wave*	1 (4.2%)	1 (0.2%)	0.102	
Abnormal EKG‡	18 (69.2%)	125 (29.2%)	<0.001	4.11 (1.7–9.9)
Age (>45ys) ‡	16 (59.3%)	183 (40%)	0.048	1.7 (0.74–3.94)
Gender male‡	18 (66.7%)	136 (29.7%)	<0.001	3.66 (1.55–8.63)

Note: All data are categorical and reported as total number (frequencies). QTc: Corrected QT Interval.

*In the ejection fraction (EF) <50% group, these parameters could not be evaluated in 2 patients, one with third degree block and other with pacemaker ventricular rhythm.

^†^This parameter could not be evaluated in one patient with pacemaker ventricular rhythm.

^‡^Gender and abnormal EKG were selected among significant variables.

The association between the three different classifications and echocardiographic, demographic and clinical variables is shown in Table 5. LVEDD, EF, and segmental contractile impairment showed a significant correlation with the Kuschnir classification (p<0.001). Diastolic dysfunction was also more frequent among patients with a worse Kuschnir classification (p = 0.005). Similar results were obtained when patients were classified by the echostage. A higher rate of CC was also associated with male and elderly patients. It is worth noting that 11.7% of the male patients had CC when defined by echocardiographic criteria, compared with 2.7% of the female patients.

**Table 5:** Echocardiographic and epidemiological variables according to Kuschnir classification, echostage classification and echocardiographic/electrocardiographic parameters.

	Kuschnir 0 N = 233	Kuschnir I N = 98	Kuschnir II N = 8	Kuschnir III N = 9	P value	Echostag I N = 302	EchostagII N = 126	EchostagIII N = 27	P value	noCC n = 302	CC n = 153	P value
Age	36 (30–44)	41 (36–47)	49.5 (45–56) (10.27)	53(38.5–68.5)	<0.001	37 (31–44)	42 (36–49)	45 (35.25–50.75)	<0.001	37 (31–44)	42 (36–49)	<0.001
Gender (male)	58 (24.9%)	42 (42.9%)	4 (50%)	5 (55.6%)	0.01	75 (24.8%)	53 (42.1%)	15 (75%)	<0.001	75 (24.8%)	71 (46.4%)	<0.001
NYHA 0	233 (100%)	98 (100%)	8 (100%)	0	<0.001	302 (100%)	125(99.2%)	19 (70%)	<0.001	302 (100%)	144 (94.%)	<0.001
Cardiomegaly	0	0	8 (100%)	8 (100%)	<0.001	1 (0.4%)	5 (5.1%)	9 (42.9%)	<0.001	1 (0.4%)	14 (11.8%)	<0.001
LVEDD	46 (43–49)	48 (43–48)	52 (46–52)	61 (53–65)	<0.001	46 (43–49)	48 (45–50)	56 (49–58)	<0.001	46 (43–49)	48.5 (45–52)	<0.001
LVESD	29 (26–31)	30 (28–33)	32 (31–34)	46 (37–52)	<0.001	29 (26–31)	30 (28–33)	37 (35–41)	<0.001	29 (26–31)	31 (29–33)	<0.001
ST	9 (8–10)	10 (9–11)	11 (9–12)	10 (9–13)	<0.001	9 (8–10)	10 (8–11)	10 (9–11)	<0.001	9 (8–10)	10 (9–11)	<0.001
PT	9 (8–10)	9 (8–10)	10 (9–11)	10 (9–11)	0.01	9 (8–10)	9 (8–10)	9.5 (9–10)	0.03	9 (8–10)	9 (8–10)	0.012
LAD	36 (33–39)	38 (34–40)	42 (37–45)	44 (40–50)	<0.001	36 (33–40)	38 (34–40)	42 (40–44)	<0.001	36 (33–42)	39 (34–41)	<0.001
EF	62 (59–66)	62 (59–66)	60 (59–65)	44 (31–51)	<0.001	62 (59–66)	62 (59–66)	53 (47–61)	<0.001	62 (59–66)	62 (57–64)	0.18
TAPSE	24 (22–26)	23 (21–26)	25 (21–29)	20 (16–27)	0.66	24 (22–26)	23 (21–26)	23.5(20–27)	0.72	24 (22–26)	23 (21–26)	0.71
E’ velocity	0.8 (0.7–0.9)	0.7(0.6–0.8)	0.6 (0.5–0.9)	0.8 (0.5–1)	0.01	0.8 (0.72–0.90)	0.75 (0.69–0.90)	0.725 (0.64–0.80)	<0.001	0.8 (0.72–0.90)	0.7 (0.67–0.88)	<0.001
DT	199 (167–225)	193 (166–240)	205 (159–213)	175 (160–239)	0.72	198 (167–222)	193.5 (167–238)	219 (179–265)	0.24	198 (167–222)	195 (168–240)	0.22
DD (normal)	189(81.5%)	72 (75.7%)	2(25%)	3 (42.9%)	0.01	245 (81.9%)	93 (76.2%)	13 (78.7%)	<0.001	245 (81.9%)	106 (72.1%)	0.04
CI	3 (1.3%)	0	1 (12.5%)	4 (44.4%)	<0.001	0	0	10 (37%)	<0.001	0	10 (6.5%)	<0.001
Positive PCR	76 (39.2%)	37 (50%)	2 (33.3%)	3 (100%)	0.08	76 (39.6%)	36 (49.3%)	6 (46.2%)	0.35	76 (39.6%)	42 (48.8%)	0.1

**Note:** categorical data are shown as total numbers (frequencies) and quantitative data are shown as median (interquartile range). CC, Chagas cardiomyopathy; NYHA, New York Heart Association Scale; LVEDD, left ventricular end-diastolic diameter; LVESD, left ventricular end-systolic diameter; ST, Septum thickness; PT, Posterior thickness; LAD, Left atrium diameter; EF, ejection fraction; TAPSE, tricuspid annular plane systolic excursion; DT, Deceleration time; CI, contractile impairment; DD, diastolic dysfunction; PCR polymerase chain reaction.

We analyzed the best combination of criteria for ruling out echocardiographic impairment using the ROC curve (age) and EKG abnormality parameter. In the case of females, being older than 45 years old or having any EKG abnormality has a negative predictive value of 100%. In the case of males you need to set the minimum age to 30 or have any EKG abnormalities to achieve the same negative predictive value. More information is shown in Table 6.

**Table 6:** Sensitivity and specificity of the selected criteria to detect echocardiographic impairment.

Criteria	Sensitivity (95%CI)	Specificity (95%CI)	Positive predictive value	Negative predictive value	Likelihood negative ratio (95%CI)	Likelihood positive ratio (95%CI)
Abnormal EKG	69.2% (48.2–85.7%)	70.6% (66–74.8%)	12.5% (7.58–19%)	97.4% (95–98.9%)	0.43 (0.24–0.77)	2.35 (1.75–3.16)
Female >45 years	44.44% (6.43–82.46)	75.47% (70.61–80.32)	4.82% (0–10.03)	97.98%(96.03–99.93)	0.74 (0.41–1.32)	1.81 (0.85–3.85)
Male >30 years	88.89% (71.59–100%)	24.26% (16.69–31.84%)	13.45% (6.9–19.99%)	94.29% (85.17–100%)	0.46 (0.12–1.75)	1.17 (0.97–1.42)
Female >45 years old or any EKG findings suggestive of CC	100% (94.44–100)	57.65 (51.96–63.34)	6.47 (2.02–10.93)	100%(99.72–100)	0	2.36 (2.07–2.69)
Male >30 years old or any EKG findings suggestive of CC	100% (97.22–100)	15.67% (9.14–22.2%)	13.74% (7.46–20.02%)	100% (97.62–100%)	0	1.19 (1.1–1.28)

**Note:** 95% CI, 95% confidence interval.

*T*. *cruzi* DNA PCR before the initiation of treatment was available in 303 patients. It resulted positive in 128 (42.2%) patients. No association was found between a positive PCR result and cardiac involvement (Table 5).

## Discussion

CC is the most feared complication of CD, accounting for the majority of fatalities and disabilities related to the disease.[2] When present in the final stages it has a higher mortality rate than other cardiomyopathies.[11] CC has been closely studied in endemic areas, but data from non-endemic countries is still lacking.

To date, several classifications have been used to better predict the risk of progression and life expectancy of patients with CD.[12,20] Classically, EKG, x-ray thorax and clinical status, still highly used due to the ease of data collection, comprised the cornerstone of the classification. Even so, echocardiography is a non-invasive imaging modality that allows clinicians to have more accurate knowledge of the condition of the heart, and has been extensively studied in an attempt to find early echocardiographic markers of cardiomyopathy[21]. Accordingly, classifications that include echocardiographic data might be more accurate, since heart arrhythmias are usually accompanied by structural heart alterations.[22]

Whether or not a patient will develop CC is probably one of the main questions clinicians dealing with CD patients have. Parameters based on laboratory biomarkers, such as brain natriuretic peptide, have been studied with optimistic but inconclusive results.[21] In this context, echocardiography and EKG studies may generate special interest as the tests are radiation-free and painless.

A low ejection fraction, enlarged left ventricle and diastolic diameter of the left ventricle are clearly associated with poor cardiac outcomes. Nevertheless, once they are present cardiac damage is widely established.[10] Bearing this in mind, others parameters like diastolic dysfunction and deceleration time have shown promising results,[21,23] despite the fact that our study does not support the use of deceleration time as an early marker of disease. On the other hand, higher proportions of diastolic dysfunction were associated with a poorer Kuschnir and echostage classification.

With respect to EKG results, many findings have been associated with poor prognosis in CC. [10,24,25] Our data show that the presence of atrial arrhythmia (including pacemaker wearers) and prolonged QRS duration were associated with abnormal echocardiography. Contrary to other, QTc interval, low voltage and ST-T abnormalities did not correlate with abnormal echocardiography.[26]

When using established criteria based on left ventricular dysfunction, segmental contractile impairment and enlargement of the left ventricle, only 5.6% of the patients had CC. In turn, seventy five percent were male and 15 out of 27 were over 45 years. This highlights the importance of age and gender to develop heart involvement in CD patients.[10,27,28] Clinicians attending *T*. *cruzi* infected patients should be aware of the peculiarities of elderly patients and closely monitor them as they grow older.[29] In accordance with previous studies carried out in non-endemic areas, low cardiac involvement in patients with CD was found.[21,30] Surprisingly, patients from the same area and with similar baseline characteristics have great differences in CC depending on whether they are studied in an endemic or non-endemic area.[26,28,31] It is probable that continuous exposure to the parasite, high rates of other cardiovascular factors and the greater mean age compared with our study population could explain such differences. Another factor to consider is that *T*. *cruzi* genotypes differ geographically, which may affect the clinical course of the patients.[32] As opposed to other studies we did not find patient with normal EKG and wall motion abnormalities.[33]

As previously described, patients with normal EKG are very unlikely to have abnormal echocardiography and poor prognosis, hence EKG is a useful, cheap and non-invasive test to initially assess cardiac involvement in CD patients.[29] A normal EKG is a marker of a good prognosis in CD patients.[14] EKG abnormalities are usually seen before the patient suffers a fatal denouement from malignant arrhythmia or heart failure. In our study, we identified 8 patients with normal EKG and abnormal echocardiography. Other studies found even a higher percentage of discordance that we did, for example Viotti et al found 66 out of 849 (7.8%) patients with CD, with normal EKG and segmental lesions on echocardiography,[9] and Carrasco et al found 87 out of 486 patients (17.9%) with CD, with normal EKG and abnormal left cineventriculogram.[34] In order to avoid missing these patients, we analyzed a combination of criteria to help us decide whether or not to perform an echocardiographic examination. Echocardiography may be spared for males under 30 and females under 45 years old with normal EKG as the likelihood of having an abnormal echocardiography is minimal. Otherwise, we recommend performing the test.

Consequently, we consider that EKG should be used for the first evaluation of the heart in CD patients, with a second step evaluation using echocardiography being done in all subjects with abnormal EKG and in all males over 30 and females over 45, irrespective of the EKG findings in non-endemic countries. Other strategies like performing an echocardiography study on all *T*. *cruzi* infected patients have been proposed,[14] but economic constraints and low profitability demand more efficient strategies. Rassi et al recommended a similar algorithm based on studies in endemic countries using EKG as the first test, and then stratifying the patients according to signs and symptoms. In this algorithm echocardiography could be spared in patients with normal EKG and patients with NYHA class III/IV. [29]

So far, the chronopathogenesis of CC remains unknown. Several hypotheses have been postulated to explain how heart damage is produced.[35] Initially, autoimmune hypotheses prevailed;[36] nowadays, however, it is accepted that parasite presence is essential for cardiac damage.[37] Indeed, direct destruction of heart tissue by parasites and uncontrolled immune response may explain heart damage.[38] Therefore, treatment of CD in the indeterminate form is recommended on the basis of reducing parasite load and, as a result, preventing CC.[9,39] However, a recent manuscript discourage from treating patients with established CC.[40] In this context, several approaches which associate *T*. *cruzi* DNA in peripheral blood with cardiac complications have been conducted with conflicting results.[7,8] Our study did not find a statistically significant association between *T*. *cruzi* DNA in peripheral blood and cardiac involvement (39.6% vs 48.8%; p = 0.1).

The strength of our study lies in its large number of well studied patients with CD, as well as in the rigorous selection criteria applied in order to avoid possible confusion factors. Additionally, our study was performed in a non-endemic country and the results may be extrapolated to other non-endemic regions.

Our study is cross-sectional and conclusions about the prognostic value of any variable cannot be drawn. Another possible limitation to the conclusions of our study is that we found a low rate of CC as well as few echocardiography abnormalities.

In summary, we studied a large number of CD patients and identified a low rate of CC. Age and sex are important and unchangeable determinants for the development of CC, and in addition to EKG abnormalities, may be taken into account when deciding whether or not to perform an echocardiography. Among echocardiographic parameters, diastolic dysfunction has been shown to be associated with poorer cardiac status, and it may be used as an early marker of CC.

## Supporting Information

S1 Checklist (DOCX)

## Abbreviations

CD: Chagas disease
EKG: Electrocardiogram
PCR: Polymerase chain reaction
CC: Chagas cardiomyopathy
DNA: deoxyribonucleic acid
2D-TTE: two dimensional transthoracic echocardiogram
ELISA: enzyme-linked immunosorbent assay
NYHA: New York Heart association
AV: atrioventricular
EF: ejection fraction
IQR: interquartile range
LVEDD: left ventricular end diastolic diameter
